# Detection of HHV-6 and EBV and Cytokine Levels in Saliva From Children With Seizures: Results of a Multi-Center Cross-Sectional Study

**DOI:** 10.3389/fneur.2018.00834

**Published:** 2018-10-05

**Authors:** Luca Bartolini, Eleonora Piras, Kathryn Sullivan, Sean Gillen, Adrian Bumbut, Cheng-Te Major Lin, Emily C. Leibovitch, Jennifer S. Graves, Emmanuelle L. Waubant, James M. Chamberlain, William D. Gaillard, Steven Jacobson

**Affiliations:** ^1^Center for Neuroscience, Children's National Medical Center, George Washington University, Washington, DC, United States; ^2^Clinical Epilepsy Section, National Institute of Neurological Disorders and Stroke, Bethesda, MD, United States; ^3^Division of Neuroimmunology and Neurovirology, National Institute of Neurological Disorders and Stroke, Bethesda, MD, United States; ^4^Neuroimmunology Unit, Santa Lucia Foundation, Rome, Italy; ^5^Emergency Medicine and Trauma Services, Children's National Medical Center, Washington, DC, United States; ^6^Multiple Sclerosis Center, University of California, San Francisco, San Francisco, CA, United States

**Keywords:** herpesvirus 6, human, inflammation, interleukin-1, saliva, epilepsy, pediatric, Epstein-Barr Virus

## Abstract

**Background and Objective:** One third of children with epilepsy are refractory to medications. Growing data support a role of common childhood infections with neurotropic viruses and inflammation in epileptogenesis. Our objective was to determine the frequency of Human Herpesvirus-6 (HHV-6) and Epstein-Barr Virus (EBV) infection and cytokine levels in saliva from children with seizures compared to healthy controls and to controls with a febrile illness without seizures.

**Methods:** In this cross-sectional multi-center study, we collected saliva from 115 consecutive children with acute seizures (cases), 51 children with a fever and no seizures or underlying neurological disease (fever controls) and 46 healthy children (healthy controls). Specimens were analyzed by a novel droplet digital PCR for HHV-6 and EBV viral DNA and a bead-based immunoassay for neuroinflammatory cytokines.

**Results:** Cases included febrile seizures (*n* = 30), acute seizures without (*n* = 53) and with fever (*n* = 4) in chronic epilepsy, new onset epilepsy (*n* = 13), febrile status epilepticus (*n* = 3), and first lifetime seizure (*n* = 12). HHV-6 DNA was found in 40% of cases vs. 37% fever controls and 35% healthy controls, with no statistically significant differences. EBV DNA was also detected with no differences in 17% cases, 16% fever controls, and 28% healthy controls. IL-8 and IL-1β were increased in saliva of 32 random samples from cases compared with 30 fever controls: IL-8 cases mean (SD): 1158.07 pg/mL (1427.41); controls 604.92 (754.04); *p* = 0.02. IL-1β 185.76 (230.57); controls 86.99 (187.39); *p* = 0.0002. IL-1β level correlated with HHV6 viral load (*p* = 0.007).

**Conclusion:** Increase in inflammatory cytokines may play a role in the onset of acute seizures and saliva could represent an inexpensive and non-invasive method for detection of viral DNA and cytokines.

## Introduction

Experimental and clinical findings suggest a crucial role of inflammation in epileptogenesis (1). Inflammation is not targeted by conventional antiepileptic drugs and may contribute to breakthrough seizures and refractory epilepsy (2). An emerging hypothesis postulates that various brain insults, including common viral infections of childhood (3), can induce acute and chronic inflammatory processes in the central nervous system (CNS) and increased permeability of the blood–brain barrier, leading to enhanced neuronal excitability, which may contribute to epileptogenesis (4, 5). Prior studies demonstrate that elevated levels of interleukin-1β (IL-1β), interleukin-6 (IL-6), interleukin-8 (IL-8), and tumor necrosis factor (TNF) are linked to neuronal hyperexcitability (1) and are found in serum and CSF of patients with epilepsy (6–10).

Primary infection with human herpesvirus-6 (HHV-6) occurs in almost 80% of children by age 2 years (11). There are two species of this virus, HHV-6A and HHV-6B, which share 90% homology at nucleotide level (12). Both species are pathogenic and show different tropism, with HHV-6B being the primary type causing disease in humans, while HHV-6A has been shown to replicate more efficiently in neural cell lines than HHV-6B (13). Previous studies detected HHV-6B DNA in blood from patients with febrile seizures, accounting for a combined frequency of 16.5% in systematic reviews of the literature (14), from 54/169 (32%) of children with febrile status epilepticus (15) and in resected epileptogenic tissue from 15/24 (62%) of mesial temporal lobe epilepsy (MTLE) cases compared to 0/14 of brain specimens from other syndromes (16). Host gene expression analysis of brain samples of patients with MTLE revealed expression of monocyte chemoattractant protein 1 (MCP-1) and glial fibrillary acidic protein (GFAP) in the HHV-6–positive vs. HHV-6–negative amygdala tissues, with positive correlation with viral load, suggesting an inflammatory process triggered by the virus (17). Inflammatory cytokines such as IL-1β and HHV-6 infection can both induce dysregulation of glutamate homeostasis in astrocytes and contribute to epileptogenesis (18, 19). Moreover, studies investigating cytokine profiles in serum and cerebrospinal fluid (CSF) from children with HHV-6 encephalopathy revealed an increase in IL-6, IL-8, Interleukin-10 (IL-10), and Matrix Metalloproteinase-9 (MMP-9), compared to healthy children (20).

The central nervous system (CNS) represents a potential site of viral latency for HHV-6 (21). Similarly to what is seen with a number of other neurotropic viruses, previous studies find that HHV-6 may enter the CNS through the olfactory pathway. The nasal cavity contains a group of specialized glial cells that have characteristics of CNS astrocytes and Schwann cells, known as olfactory-ensheathing cells (OEC). HHV-6 has a tropism for glial cells and can replicate *in vitro* in OEC. Infected OEC showed significant increase in production of IL-6, chemokine ligand-1 (CCL-1) and chemokine ligand-5 (CCL-5) (21). Peripherally, another proposed site of latency is in the tonsils, where HHV-6 has been shown to be actively shed in saliva (22). Interestingly, the frequency of detection of HHV-6 DNA was not different between nasal mucous and saliva (21), supporting the notion that saliva is an *in-vivo* reservoir for HHV-6. Saliva is a useful clinical specimen to collect and represents a non-invasive and low-cost method of obtaining measures of inflammation (23) and viral infection, particularly in children where blood is often difficult to obtain. Saliva has been successfully utilized in pathological conditions including cystic fibrosis (24), but not in epilepsy.

Similarly, Epstein-Barr Virus (EBV) is observed in children with acute seizures. Twenty-one (6%) of 216 children in the Encephalitis Registry at University of Toronto had positive EBV serology and/or positive PCR, and 48% presented with seizures (25). In a large Polish registry including 194 children diagnosed with EBV infection with positive serology, febrile seizures occurred in 3.1% of cases younger than 5 years (26). Seizures during the course of EBV neuroinfection have been described as generalized tonic-clonic (27), focal (28), convulsive (29), and nonconvulsive (30) status epilepticus, and myoclonic seizures (31). Expression of IL-6 and IL-1β was shown to be strongly enhanced in interfollicular areas of tonsils of children with infectious mononucleosis (32), suggesting that EBV infection results in the expression of specific cytokine genes in EBV-infected and neighboring cells, but little is known on the proinflammatory cytokine profiles in cases of neurological manifestation of EBV infection.

The aim of this study is to determine the frequency of HHV-6 and EBV infection and cytokine levels in saliva from children with seizures and compare with children with a febrile illness without seizures and in healthy children. We hypothesized that HHV-6 and EBV viral infection is more frequent in children with seizures. Our secondary hypothesis was that inflammatory cytokines are increased in saliva of children with seizures.

## Methods

In this cross-sectional study, we enrolled three cohorts of children between March 2016 and February 2017: 1. Children aged 1 month −18 years presenting at Children's National Medical Center (CNMC) within 24 h of a single or multiple seizures of any etiology or duration (cases); 2. Children aged 1 month −18 years presenting at CNMC for evaluation of a fever (≥37.8°C oral or ≥ 38.0°C rectal) without seizures or prior neurological condition (fever controls); 3. A cohort of 46 healthy children aged 7–18 years (healthy controls) that were enrolled at University of California San Francisco and had saliva collected with a different method that consisted of a mouth rinse as previously described (33) and frozen upon collection. Saliva from these children was analyzed at NINDS following the same protocol for DNA extraction and ddPCR, using the same materials and equipment. Internal validation in our laboratory showed no difference in viral detection rate and viral load when comparing the two methods in healthy volunteers (Supplementary Table 1).

Children with primary or acquired immunodeficiency or taking immunosuppressant agents were excluded.

We collected saliva utilizing a validated pediatric swab (SalivaBio Oral Swab, Salimetrics). Samples were frozen at−80°C immediately after collection and shipped on ice to the National Institute of Neurological Disorders and Stroke (NINDS) for analysis. After thawing the samples, they were centrifuged at 2,300 g for 10 min and DNA was extracted from 200 μL saliva utilizing a commercially available kit (DNeasy Blood and Tissue Kit, Qiagen). Viral DNA in saliva was quantified in all three cohorts using a novel digital droplet PCR (ddPCR) platform that was recently shown to be a precise and reliable method for virus DNA load quantification and for detection of viral mutants in the target gene sequence (34). Primers from highly conserved regions were selected for HHV-6 (u57) and EBV (lmp1): NC_001664 for HHV-6A (strain U1102), NC_000898 for HHV-6B (strain Z29) and NC_007605.1 for EBV (strain B95-8). Ribonuclease P protein subunit P30 (RPP30) was used as a cellular housekeeping gene.

Undiluted saliva from randomly selected 32 cases and 30 fever controls was analyzed for cytokine levels with a bead-based immunoassay (BD Biosciences CBA Human Inflammatory Cytokines Kit) that included IL-8, IL-1β, IL-6, IL-10, TNF, and IL-12p70 utilizing a BD Biosciences LSR II flow cytometer.

Samples were handled following standard biosafety procedures, including appropriate hand washing, use of protective equipment such as gloves and gowns, careful manipulation of specimens to avoid spilling and contamination, careful manipulation, and disposal of sharps, decontamination of laboratory equipment to avoid contamination.

Medical records were reviewed for clinical, laboratory, imaging and EEG results, if available. Study data were collected and managed using a password-protected database (REDCap electronic data capture tools hosted at CNMC).

Written informed consent was obtained from parent or legal guardian and written assent from the patient, when applicable. Children's National Medical Center and University of California San Francisco Institutional Review Boards approved the study.

The raw data supporting the conclusions of this manuscript will be made available by the authors, without undue reservation, to any qualified researcher.

### Statistical analysis

The primary outcomes were frequency of detection of HHV-6 and EBV DNA and cytokine levels in cases vs. controls. Statistical analysis was conducted utilizing R version 3.4.1. For our primary outcome, we utilized Pearson Chi-squared test and Fisher's exact test to disprove the null hypothesis that frequency of viral DNA detection is the same in cases and controls. For our secondary outcome, we tested the null hypothesis that cytokine levels are the same in cases and controls by means of Wilcoxon rank-sum test. Secondary analyses included: 1. non-parametric analysis of covariance (ANCOVA) to test differences in viral load in cases vs. fever controls and non-age-matched healthy controls; 2. Pearson's correlation to analyze the possible influence of fever grade and age on cytokine levels; 3. polyserial correlation to analyze the possible influence of viral load on cytokine levels. Cytokine levels below the threshold of quantification were assumed as mean between 0 and the lower limit of quantification based on the standard curve for each analyte. A *p* < 0.05 was considered significant.

## Results

### Demographic and clinical data

We enrolled 115 consecutive cases [mean ± standard deviation (SD) 6.3 ± 5.1 years, 67% male] and 51 fever controls (5.9 ± 5.5 years, 53% male). Table 1 shows diagnoses and age of all cases and controls. The majority of cases (*n* = 90, 78%) had seizures lasting <5 min and 36 cases (31%) had more than one seizure. 17 controls (33%) presented with one or more upper respiratory infection (URI) symptoms (sneezing, congestion, rhinorrhea), 17 (33%) had one or more lower respiratory infection (LRI) symptoms (cough, labored breathing, tachypnea). Mean temperature was 38.9°C (SD 0.67) and 31 (61%) had had fever for more than 24 h at time of enrollment. Thirty seven cases (32%) had a fever (mean 38.8°C, SD 0.57) and 34 (29%) had URI symptoms; only 3 cases (3%) had LRI symptoms.

**Table 1:** Diagnosis, demographic data and frequency of detection of viral DNA in cases and controls.

	**n**	**Age (y) M (SD)**	**Fever *n* (%)**	**HHV-6A *n* (%)**	**HHV-6B *n* (%)**	**EBV *n* (%)**	**Co-det *n* (%)**
All cases	115	6.3 (5.1)	37 (32)	1 (1)	46 (40)	20 (17)	8 (7)
Febrile seizure	30	2.1 (1.0)	30 (100)	0	15 (50)	5 (17)	2 (7)
Epilepsy seizure without fever	53	8.5 (5.1)	0	1 (2)	22 (41)	10 (19)	4 (7)
Epilepsy seizure with fever	4	4.0 (1.2)	4 (100)	0	2 (50)	0	0
New epilepsy	13	5.9 (3.7)	0	0	2 (15)	3 (23)	2 (15)
Status epilepticus	3	8.6 (3.1)	3 (100)	0	1 (33)	1 (33)	0
1st acute seizure	12	7.9 (6.8)	1 (8)	0	4 (33)	1 (8)	0
All fever controls	51	5.9 (5.5)	51 (100)	0	19 (37)	8 (16)	4 (8)
Fever controls <4 years	24	1.4 (1.2)	24 (100)	0	8 (33)	2 (8)	1 (4)
UCSF healthy controls	46	14.1 (2.8)	0	0	16 (35)	13 (28)	7 (15)

*y, years; M, mean; SD, standard deviation; Co-det, co-detection of HHV-6B and EBV*.

Brain MRI was available for 55 cases (48%), of which 7 had first acute seizures (28% abnormal), 35 had acute seizures in chronic epilepsy (60% abnormal), 9 had new onset epilepsy (22% abnormal), 2 had febrile seizures (both normal) and 2 had status epilepticus (both abnormal). Abnormal findings included structural anomalies/epileptogenic foci. EEG was available for 77 cases (67%), of which 51 (66%) were abnormal due to the presence of slowing (*n* = 10), epileptiform discharges (*n* = 20) or both (*n* = 21).

Healthy controls without fever consisted of 46 healthy, asymptomatic, older children aged 7–18 years (mean 14.1 years, SD 2.8).

### Viral PCR results

**Figure 1:**
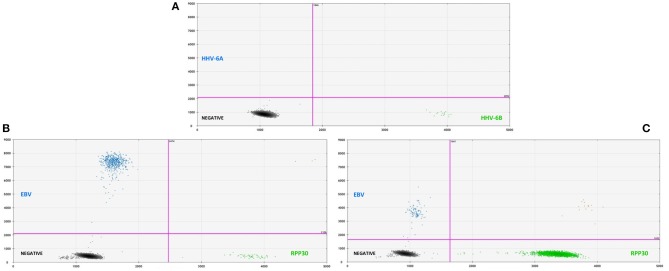
**(A**, top) Representative two-dimensional ddPCR plot from a 2 years old boy with febrile seizure who tested positive for HHV-6B (green dots, right lower quadrant) in saliva. The x-axis displays the amplitude of fluorescence, corresponding to HHV-6B, while the y-axis displays the fluorescence amplitude of HHV-6A. Different probes were used to distinguish between HHV-6A (FAM) and HHV-6B (VIC). **(B**, bottom left) Representative plot for the detection of EBV DNA (blue dots, left upper quadrant) in saliva from a 14 years-old young man with an acute seizure in the context of chronic epilepsy. **(C**, bottom right) Example of low-amplitude lmp1 mutation in a 8 years-old child with epilepsy and acute seizures. ddPCR is a novel technique that allows for the direct absolute quantification of a target gene in a given sample, providing a precise and reliable method for quantification of proviral load, particularly when applied to samples with low numbers of cells.

Overall, 46/115 cases of children with seizures of any etiology tested positive for HHV-6B DNA in saliva (40%) (Table 1). Of all 115 cases, 30 presented with febrile seizures and 50% were positive for HHV-6B. While this frequency was higher than age-matched controls with fever or older healthy controls (50% vs. 37% vs. 35%), the difference did not reach significance. The rate of detection of EBV DNA in saliva was homogeneously lower across groups: 17% in all children with seizures, 16% in fever controls, and 28% in older healthy controls (Table 1). Of the 53 children with epilepsy and no fever presenting with an acute seizure, 22 (41%) tested positive for HHV-6B, 1 for HHV-6A (co-infected with HHV-6B and only HHV-6A positive case in the study) and 10 (19%) for EBV. Representative ddPCR plots for HHV-6 and EBV are shown in Figures 1A,B.

Analysis of HHV-6B and EBV viral load adjusted for age showed no significant difference across groups. Similarly, we did not observe differences in viral loads in patients with single vs. multiple or <5 min vs. ≥ 5 min seizures.

In addition to serving as a highly reliable tool for quantifying herpesvirus DNA, ddPCR was also shown to be useful for detecting viral mutations in the target gene. As described previously, the fluorescence amplitude of the positive droplets reflects primer/probe binding and is specific to each primer/probe set. This is exemplified in Figure 1C, in which the amplitude of the EBV lmp1 probe is lower for an 8 years-old child with epilepsy and acute seizures compared with prototype EBV in Figure 1B. In our population of children with epilepsy presenting with acute seizures (*n* = 53), 10 had EBV DNA in saliva and of these, 4 had a low-amplitude lmp1 mutation (40%). Three children with a new diagnosis of epilepsy were infected by EBV and we detected both HHV-6B and EBV DNA in two of them, both with low-amplitude lmp1 mutation. Similarly, 5/13 (38%) of healthy children who were positive for EBV had low-amplitude lmp1 mutation.

### Cytokine analysis results

**Figure 2:**
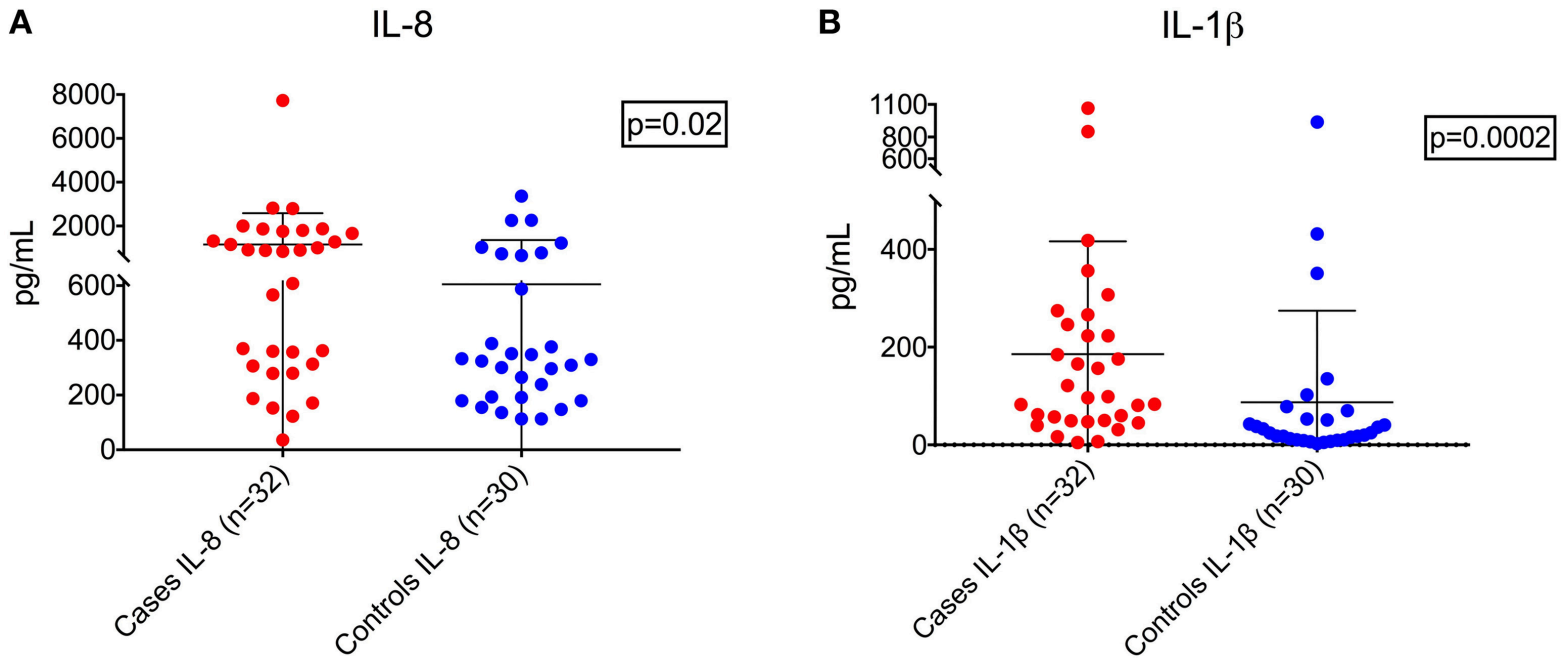
Higher levels of IL-8 **(A**, left; Wilcoxon rank-sum test, W = 641, *p* = 0.02) and IL-1β **(B**, right; Wilcoxon rank-sum test, W = 738, *p* = 0.0002) in children with seizures vs. controls with fever.

Demographic and clinical characteristics and cytokine profiles of the random sample of age-matched cases and fever controls that was analyzed are summarized in Tables 2–4. Compared to fever controls and in agreement with our hypothesis, saliva from children with seizures exhibited higher levels of IL-8 (median cases = 861.5 pg/mL, median fever controls = 327.3 pg/mL, W = 641, *p* = 0.02) and IL-1β (median cases = 97.48 pg/mL, median fever controls = 24.63 pg/mL, W = 738, *p* = 0.0002) (Figures 2A,B). The two groups had similar frequency of HHV-6B infection. IL-6, IL-10, TNF, and IL-12p70 profiles were not different between groups and were overall low.

**Table 2:** Demographic characteristics of a randomly selected cohort of cases and controls whose saliva was analyzed for cytokine levels.

	**Cases, *n* = 32**	**Controls, *n* = 30**	***P*-value**
Age, y (M, SD)	6.8 (5.3)	5.5 (4.6)	n.s.
Sex, M (*n*, %)	18 (56%)	17 (57%)	n.s.
Fever (*n*, %)	9 (28%)	30 (100%)	<0.0001
Fever grade, °C (M, SD)	38.8 (0.4)	38.9 (0.6)	n.s.
HHV-6B positive	12 (37%)	13 (43%)	n.s.

*M, mean; SD, standard deviation; n.s., not significant*.

**Table 3:** Clinical characteristics of the 32 randomly selected cases whose saliva was analyzed for cytokine levels.

	**All cases**	
EEG obtained (*n*, %)	24 (75%)	
Abnormal EEG (*n*, % of EEGs)	18 (75%)	
Brain MRI obtained (*n*, %)	18 (56%)	
Abnormal brain MRI (*n*, % of MRIs)	4 (22%)	
Seizure type: focal onset with altered awareness (*n*, %)	15 (47%)	
Seizure type: generalized onset (*n*, %)	17 (53%)	
	**HHV-6 positive**	**HHV-6 negative**
*N*, %	12 (37%)	20 (63%)
**Diagnosis**		
Febrile seizure (*n*, %); Age, y (M, SD)	5 (42%); 1.9 (0.4)	2 (10%); 2.1 (0.6)
Acute seizure in epilepsy (*n*, %); Age, y (M, SD)	6 (50%); 8.7 (5.3)	11 (55%); 6.9 (5.8)
1st acute seizure (*n*, %); Age, y (M, SD)	1 (8%); 8.4	3 (15%); 10.7 (4.6)
New diagnosis epilepsy; Age, y (M, SD)	–	4 (20%); 8.8 (1.8)

*M, mean; SD, standard deviation; y, years*.

**Table 4:** Cytokine levels in saliva of a randomly selected cohort of cases and controls.

	**Cases (*n* = 32)**	**Controls (*n* = 30)**	
**Cytokine**	**M (SD) pg/mL**	**M (SD) pg/mL**	***P*-value**
Interleukin-8	1158.07 (1427.41)	604.93 (754.05)	0.02
Interleukin-1β	185.76 (230.57)	86.99 (187.39)	0.0002
Interleukin-6	42.06 (70.85)	36.18 (76.69)	n.s.
Interleukin-10	0.55 (0.66)	0.40 (0.47)	n.s.
Tumor Necrosis Factor	1.04 (1.10)	0.95 (0.87)	n.s.
Interleukin-12p70	1.57 (1.10)	1.87 (1.31)	n.s.

*M, mean; SD, standard deviation; n.s., not significant*.

Because we found higher levels of cytokines, we were then interested in determining possible associations with clinical and demographic variables.

**Figure 3:**
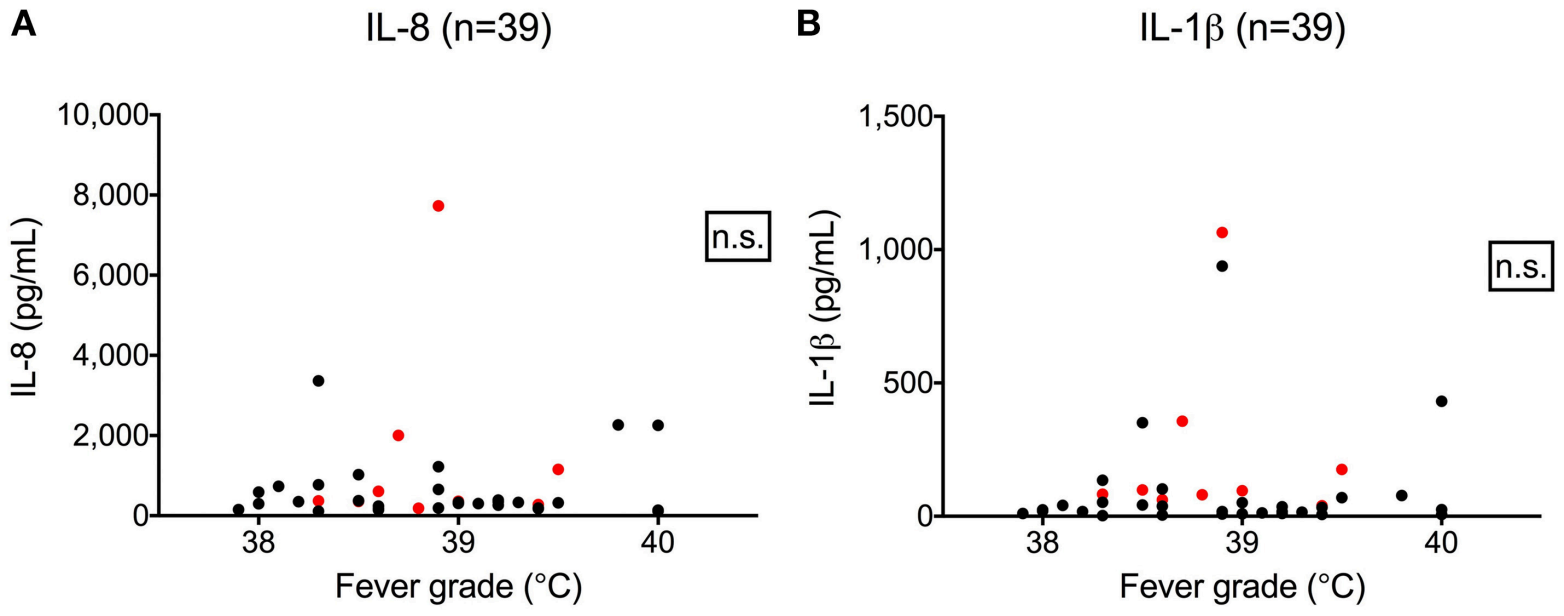
Lack of correlation between fever grade and IL-8 **(A**, left; Pearson's correlation, *r* = 0.03, 95% CI –0.2804–0.3498, *p* = 0.81) and IL-1β level **(B**, right; Pearson's correlation, *r* = 0.06, 95% CI –0.2611–0.3679, *p* = 0.72). Red dots = cases; black dots = fever controls.

**Figure 4:**
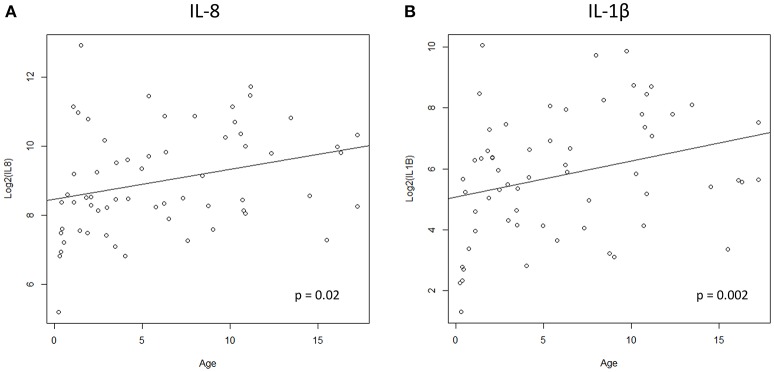
Positive correlation between age and IL-8 **(A**, left; Pearson's correlation, *t* = 2.3, 95% CI 0.04–0.50, *p* = 0.02) and IL-1β **(B**, right; Pearson's correlation, *t* = 2.3, 95% CI 0.04–0.50, *p* = 0.002).

**Figure 5:**
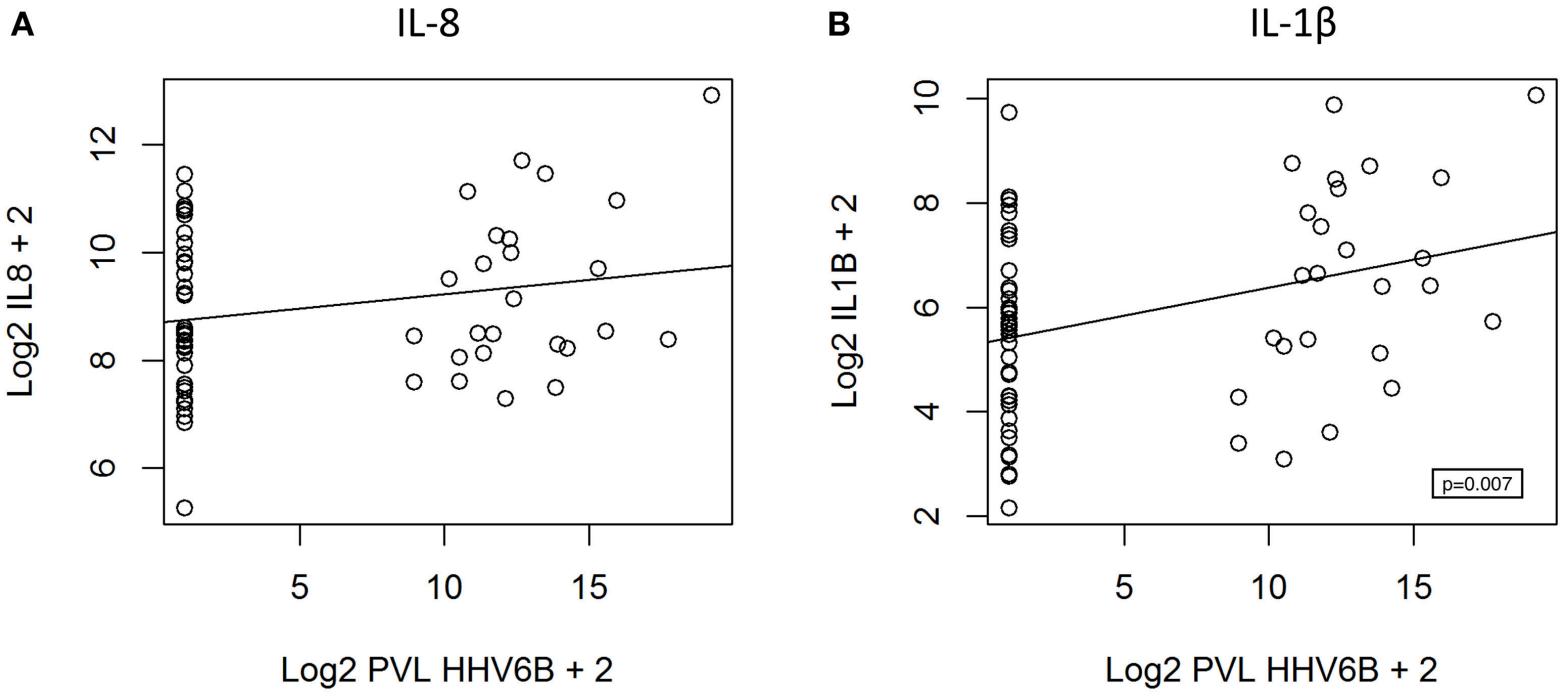
Positive correlation between HHV-6B viral load and IL-1β level **(B**, right; polyserial correlation, cor = 0.34, *p* = 0.007) and absence of correlation for IL-8 **(A**, left; polyserial correlation, cor = 0.21, *p* = 0.09). PVL = proviral load.

We hypothesized that cytokine levels in saliva from 39 children that had a fever (including 9 cases and 30 controls) would be correlated with temperature, but this was not the case (Figure 3). Similarly, we did not observe a correlation between duration of fever and cytokine levels. However, we did find a positive correlation between age and IL-8 (*t* = 2.3, 95% CI 0.04–0.50, *p* = 0.02) and IL-1β (*t* = 2.3, 95% CI 0.04–0.50, *p* = 0.002) (Figure 4) but not for the other cytokines. Since we had found that children with febrile seizures had high frequency of HHV-6B DNA detection, and it has been previously shown that this virus induces cytokine expression (35), we next asked if there was a correlation between virus detection and cytokine levels in saliva. Of 25 children (cases and fever controls) that tested positive for HHV-6B DNA in saliva, we observed a positive correlation between viral load and cytokine level for IL-1β (cor = 0.34, *p* = 0.007) but no difference for IL-8 (cor = 0.21, *p* = 0.09) (Figure 5). Breakdown of diagnoses and ages is included in Table 2 and shows no difference in HHV-6B positive vs. HHV-6B negative children. Cytokine profiles did not differ in children with single vs. multiple or <5 min vs. ≥ 5 min seizures.

## Discussion

In our study, we employed a novel ddPCR technique to assess presence of HHV-6 and EBV viral DNA in saliva and we did not find a significant difference in frequency of viral infection comparing children with seizures and those with acute febrile illness or older healthy children. Consistent with our secondary hypothesis, we demonstrated higher levels of IL-8 and IL-1β in saliva of children with seizures compared with age-matched fever controls and showed a positive correlation between HHV-6 viral load and IL-1β level in saliva.

Previous studies reported a highly variable frequency of seizures in children where HHV-6 DNA was detected in blood, ranging from 8 to 100% (36–38). This broad range is potentially the result of several factors, including different sensitivity of PCR techniques, different assays, DNA extraction methods, population tested, and interpretation may be limited due to lack of control populations, and often times lack of testing for co-infections with other viruses. Studies using blood and pediatric controls with a febrile illness (39) agree with our findings in saliva, and reported no difference in frequency of HHV-6 DNA detection in children with vs. children without seizures (35% vs. 50%, respectively). These results support our hypothesis that saliva may be a good surrogate for blood testing. Our data are also in agreement with a large population-based study that followed 277 infants from birth until age 2 years, analyzed serial saliva (and when available blood) samples by PCR, and showed a cumulative incidence of HHV-6 infection of 77% by 24 months of age—but none of these children had seizures (11).

The FEBSTAT study detected by qPCR HHV-6B blood viremia in 54 of 169 children (32%) aged 1 month −5 years presenting with febrile status epilepticus (15). The study used historical controls and reported an HHV-6 DNA detection rate of 9.7% in young children with acute febrile illness (40), and concluded that HHV-6 infection is commonly associated with febrile status epilepticus. Our results in saliva, with a novel ddPCR technique, highlight that the frequency of HHV-6 DNA detection in children with acute febrile illness may be higher (37%), and comparable to the FEBSTAT observations in blood from children with status epilepticus. This difference needs to be interpreted with caution, considering the different biological compartments that were analyzed, but highlights the need for further exploration with simultaneous blood and saliva samples from cases and controls. Interestingly, previous studies showed similar results when comparing HHV-6 DNA detection in saliva and blood from febrile infants (41), healthy adults (42, 43) and patients with drug reaction with eosinophilia and systemic symptoms (DRESS) (44).

We report the presence of HHV-6A DNA in one immunocompetent child with epilepsy who presented with an acute seizure. This observation is significant since HHV-6A is thought to be acquired later in life and infection is often asymptomatic (13), with the exception of Sub-Saharan Africa, where it has been described as the prevalent viral pathogen responsible for a febrile illness in immunocompromised hosts and infants (45). While previous studies had detected HHV-6A DNA in blood from adult patients with multiple sclerosis (46) and rhombencephalitis (47), its role in epilepsy has not been established. Previous studies on blood from children with febrile status epilepticus (15) and brain tissue from MTLE patients (16) did not identify acute HHV-6A infection in their participants.

Neurological complications of EBV infection are rare, however they have been reported in up to 7% of hospitalized patients for mononucleosis (48). Previous large series have shown an association with acute seizures, mostly symptomatic, often in the context of acute encephalitis. The Hospital for Sick Children encephalitis registry found evidence of acute EBV infection in 9.7% of children admitted with acute encephalitis, often without infectious mononucleosis syndrome (25). The role of this virus in epileptogenesis is less well established (49). Diffuse slowing on electroencephalogram (EEG) in patients with infectious mononucleosis in the absence of significant clinical neurologic manifestations has been reported (50). The mechanisms by which the virus causes neurological manifestations are incompletely understood, but may involve a disrupted immune response toward the infection (51). As an example, an expansion in humoral response to EBNA1 was associated with an increased risk of developing multiple sclerosis (52). The low frequency of detection of EBV DNA in saliva from cases and fever controls compared to older healthy controls can probably be attributed to the fact that EBV infection is uncommon before the age of 5 years (53–55). These differences will need to be further elucidated by a larger cohort of children, preferably including saliva, blood, and ideally CSF.

Another aim of our investigation was to explore a potential association between onset of seizures in children and cytokine levels in saliva. Overexpression of several cytokines, including IL-1β and IL-8, has been previously reported in brain tissue, serum and CSF of patients with epilepsy (4), but data on saliva are lacking. Increase in these inflammatory mediators may be the result of seizure activity itself (1), but experimental findings in mice also suggest that IL-1β, TNF, and IL-6 contribute to seizure generation and recurrence (56, 57). As a consequence, several triggers for inflammation, including viruses, may initiate a self-perpetuating cascade of events that may ultimately lead to epileptogenesis (58). Previous pediatric studies that analyzed blood and CSF of children with seizures and animal models have described a potential role of IL-1β in the genesis of seizures and later development of epilepsy (59–63). Our findings support these observations, and for the first time show that IL-1β and IL-8 expression in saliva could be a biomarker of acute activation of the neuroendocrineimmune system (64) in response to the acute stress generated by seizures in children, as increased levels were not present in the control population which had a similar frequency of HHV-6B infection. While acute seizures can be the result of different underlying etiologies, identification of a potential novel biomarker in saliva may contribute to the overall understanding of the inflammatory response in acute seizure generation and recurrence.

Similar to our findings in saliva, high levels of IL-8 and IL-1β were recently described in blood from a cohort of children initially enrolled in the FEBSTAT study (10). We demonstrated that level of these cytokines is not influenced by fever, and therefore may be directly related to seizure activity. The trigger for cytokine dysregulation in children with epilepsy remains to be elucidated, but the role of infection with HHV-6 may be significant, particularly in cases of febrile seizures, where the frequency of viral detection is high. In a small sample, we observed a positive correlation between HHV-6B viral load and IL-1β levels in saliva. While the same correlation could not be demonstrated for IL-8, these observations require further exploration with a larger cohort of patients and could potentially contribute to our understanding of how neurotropic viruses such as HHV-6 may participate in cytokine dysregulation and epileptogenesis. While our cohorts analyzed for cytokine levels were age-matched, we observed a positive correlation between age and IL-8 and IL-1β levels in saliva. Previous studies in blood analyzing the influence of age on cytokine levels report mixed results: IL-6, IL-10, and TNF-alpha were studied in 79 healthy children stratified by age and showed that IL-6 is significantly lower during the second year of life than later (65). Another study analyzed 22 cytokines in healthy cohorts of 55 patients above age 65 years and 55 patients under the age of 45; soluble CD40 ligand (sCD40L) and transforming growth factor alpha (TGF-α) levels were significantly higher in the elderly patients, whereas granulocyte colony-stimulating factor (G-CSF), granulocyte-monocyte colony-stimulating factor (GM-CSF), and monocyte chemoattractant protein-1 (MCP-1) levels were lower (66). A large study showed significant correlation between age and IL-6 from age 25 to 80 years (67). Another research showed that age positively correlated with TNF-alpha expression in healthy volunteers, kidney transplant patients and dialysis patients and with IL-2 expression in healthy volunteers and kidney-transplant patients (68). Data in saliva and in children are lacking and larger pediatric studies are needed to confirm the findings of the current preliminary study.

Limitations of this exploratory study include the small sample size of cases and fever controls that were analyzed for cytokine levels and lack of cytokine analysis in healthy children. The latter cohort was older than cases and fever controls. Cytokine levels may be influenced by time from last seizure and further studies are needed to define how time interval contributes to this difference. Because saliva was the only biological compartment analyzed, serological analysis for HHV-6B and EBV was not performed. It is possible that these data may not reflect virus representation in the serum, CSF or brain tissue. The data generated from these preliminary observations served as the basis for an ongoing study where we are analyzing simultaneous saliva and blood samples from children with acute seizures and age-matched healthy controls for detection of viral DNA and a broader cytokine panel. As in most cross-sectional studies, despite all efforts to minimize the possibility of selection bias, certain subgroups of children with acute seizures may have been under—or over-represented, compared to the background population.

Future larger studies are needed to help elucidate how cytokine expression is influenced by time from seizure onset, duration of seizure, seizure type, etiology (if known), and prospectively to monitor how these profiles change over time in children who for example presented with a first unprovoked seizure and later develop epilepsy or in those whose epilepsy becomes medically refractory over time. These studies may help in the design and development of novel therapies targeting neuroinflammation rather than symptomatically treating seizures.

## Conclusion

Our study demonstrated increased levels of inflammatory cytokines in saliva of children with seizures, collected with an inexpensive and non-invasive technique. These results will require validation with a larger cohort of patients and simultaneous healthy controls, analyzing a broader panel of viruses and cytokines both in saliva and blood.

Our preliminary findings may represent a first step toward highlighting the importance of a novel potential biomarker that could help stratify children with seizures who might best benefit from anti-inflammatory treatments and a non-EEG measure of the recent experience of a seizure.

## Ethics statement

This study was carried out in accordance with the recommendations of Children's National and University of California San Francisco IRB with written informed consent from all subjects. All subjects gave written informed consent in accordance with the Declaration of Helsinki and written assent when applicable. The protocol was approved by Children's National and University of California San Francisco IRB.

## Author contributions

LB designed the study, contributed to data analysis and interpretation, drafting and revising of the manuscript, and is responsible for final approval of the manuscript. EP, C-TL, and EL contributed to data analysis and interpretation, drafting and revising of the manuscript. KS, SG, and AB contributed to data collection and drafting, and revising of the manuscript. JG and EW contributed to data collection, analysis and interpretation, and drafting and revising of the manuscript. JC, WG, and SJ contributed to study design, data analysis and interpretation, and drafting and revising of the manuscript.

### Conflict of interest statement

The authors declare that the research was conducted in the absence of any commercial or financial relationships that could be construed as a potential conflict of interest.

## References

[1] Vezzani A French J Bartfai T Baram TZ. The role of inflammation in epilepsy. Nat Rev Neurol. (2011) 7:31–40. 10.1038/nrneurol.2010.178 21135885 PMC3378051

[2] van Vliet EA Aronica E Vezzani A Ravizza T. Neuroinflammatory pathways as treatment targets and biomarker candidates in epilepsy: emerging evidence from preclinical and clinical studies. Neuropathol Appl Neurobiol. (2018) 44:91–111. 10.1111/nan.12444 28977690

[3] Theodore WH. Epilepsy and viral infections. Epilepsy Curr. (2014) 14:35–42. 10.5698/1535-7511-14.s2.35 24955074 PMC3966643

[4] Bartolini L Libbey JE Ravizza T Fujinami RS Jacobson S Gaillard WD. Viral triggers and inflammatory mechanisms in pediatric epilepsy. Mol Neurobiol. (2018) 10.1007/s12035-018-1215-5. [Epub ahead of print]. 29978423 PMC7416551

[5] Vezzani A Fujinami RS White HS Preux PM Blümcke I Sander JW. Infections, inflammation and epilepsy. Acta Neuropathol. (2016) 131:211–34. 10.1007/s00401-015-1481-5 26423537 PMC4867498

[6] Henshall DC Clark RS Adelson PD Chen M Watkins SC Simon RP. Alterations in bcl-2 and caspase gene family protein expression in human temporal lobe epilepsy. Neurology (2000) 55:250–7. 10.1212/WNL.55.2.250 10908900

[7] Kan AA van Erp S Derijck AA de Wit M Hessel EV O'Duibhir E. Genome-wide microRNA profiling of human temporal lobe epilepsy identifies modulators of the immune response. Cell Mol Life Sci. (2012) 69:3127–45. 10.1007/s00018-012-0992-7 22535415 PMC3428527

[8] Ravizza T Boer K Redeker S Spliet WG van Rijen PC Troost D The IL-1b system in epilepsy-associated malformations of cortical development. Neurobiol Dis. (2006) 24:128–43. 10.1016/j.nbd.2006.06.003 16860990

[9] van Vliet EA Aronica E Gorter JA. Role of blood-brain barrier in temporal lobe epilepsy and pharmacoresistance. Neuroscience (2014) 277:455–73. 10.1016/j.neuroscience.2014.07.030 25080160

[10] Gallentine WB Shinnar S Hesdorffer DC Epstein L Nordli DR Lewis DV. Plasma cytokines associated with febrile status epilepticus in children: a potential biomarker for acute hippocampal injury. Epilepsia (2017) 58:1102–11. 10.1111/epi.13750 28448686 PMC5482499

[11] Zerr DM Meier AS Selke SS Frenkel LM Huang ML Wald A. A population-based study of primary human herpesvirus 6 infection. N Engl J Med. (2005) 352:768–76. 10.1056/NEJMoa042207 15728809

[12] Zerr DM. Human herpesvirus 6: a clinical update. Herpes (2006) 13:20–4. 16732999

[13] De Bolle L Naesens L De Clercq E. Update on human herpesvirus 6 biology, clinical features, and therapy. Clin Microbiol Rev. (2005) 18:217–45. 10.1128/CMR.18.1.217-245.2005 15653828 PMC544175

[14] Millichap JG Millichap JJ. Role of viral infections in the etiology of febrile seizures. Pediatr Neurol. (2006) 35:165–72. 10.1016/j.pediatrneurol.2006.06.004 16939854

[15] Epstein LG Shinnar S Hesdorffer DC Nordli DR Hamidullah A Benn EK. Human herpesvirus 6 and 7 in febrile status epilepticus: the FEBSTAT study. Epilepsia (2012) 53:1481–8. 10.1111/j.1528-1167.2012.03542.x 22954016 PMC3442944

[16] Fotheringham J Donati D Akhyani N Fogdell-Hahn A Vortmeyer A Heiss JD. Association of human herpesvirus-6B with mesial temporal lobe epilepsy. PLoS Med. (2007) 4:e180. 10.1371/journal.pmed.0040180 17535102 PMC1880851

[17] Kawamura Y Nakayama A Kato T Miura H Ishihara N Ihira M. Pathogenic role of human herpesvirus 6B infection in mesial temporal lobe epilepsy. J Infect Dis. (2015) 212:1014–21. 10.1093/infdis/jiv160 25840441

[18] Vezzani A. Epilepsy and inflammation in the brain: overview and pathophysiology. Epilepsy Curr. (2014) 14:3–7. 10.5698/1535-7511-14.s2.3 24955068 PMC3966641

[19] Fotheringham J Williams EL Akhyani N. Human herpesvirus 6 (HHV-6) induces dysregulation of glutamate uptake and transporter expression in astrocytes. J Neuroimmune Pharmacol. (2008) 3:105–16. 10.1007/s11481-007-9084-0 18247129

[20] Kawabe S Ito Y Ohta R Sofue A Gotoh K Morishima T. Comparison of the levels of human herpesvirus 6 (HHV-6) DNA and cytokines in the cerebrospinal fluid and serum of children with HHV-6 encephalopathy. J Med Virol. (2010) 82:1410–5. 10.1002/jmv.21808 20572074

[21] Harberts E Yao K Wohler JE Maric D Ohayon J Henkin R. Human herpesvirus-6 entry into the central nervous system through the olfactory pathway. Proc Natl Acad Sci USA. (2011) 108:13734–9. 10.1073/pnas.1105143108 21825120 PMC3158203

[22] Caserta MT Mock DJ Dewhurst S. Human herpesvirus 6. Clin Infect Dis. (2001) 33:829–33. 10.1086/322691 11512088

[23] Granger DA Johnson S Salivary biomarkers. In: Gellman MD Turner RJ, editors. Encyclopedia of Behavioral Medicine. New York, NY: Springer New York (2013). p. 1697–705.

[24] Nie S Zhang H Mayer KM Oppenheim FG Little FF Greenberg J. Correlations of salivary biomarkers with clinical assessments in patients with cystic fibrosis. PLoS ONE (2015) 10:e0135237. 10.1371/journal.pone.0135237 26258476 PMC4530931

[25] Doja A Bitnun A Ford Jones EL Richardson S Tellier R Petric M Pediatric Epstein-Barr Virus-associated encephalitis: 10-year review. J Child Neurol. (2006) 21:384–91. 10.1177/08830738060210051101 16901443

[26] Mazur-Melewska K Brenska I Jonczyk-Potoczna K Kemnitz P Pieczonka-Ruszkowska I Mania A. Neurologic complications caused by Epstein-Barr Virus in pediatric patients. J Child Neurol. (2016) 31:700–8. 10.1177/0883073815613563 26511720

[27] Mozes B Pines A Werner D Kaplinsky N Frankl O. Grand-mal as the major presenting symptom of infectious mononucleosis. J Neurol Neurosurg Psychiatry (1984) 47:569–70. 10.1136/jnnp.47.5.569 6429286 PMC1027844

[28] Blackwood RA. Sudden onset focal seizures in a 27-month-old boy. Pediatr Infect Dis J. (1992) 11:596–99. 1326742

[29] Russell J Fisher M Zivin JA Sullivan J Drachman DA. Status epilepticus and Epstein-Barr virus encephalopathy. Diagnosis by modem serologic techniques. Arch Neurol. (1985) 42:789–92. 10.1001/archneur.1985.04210090053015 2992428

[30] Greco F Cocuzza MD Smilari P Sorge G Pavone L. Nonconvulsive status epilepticus complicating epstein-barr virus encephalitis in a child. Case Rep Pediatr. (2014) 2014:547396. 10.1155/2014/547396 24744940 PMC3972953

[31] Bassan H Bloch AM Mesterman R Assia A Harel S Fattal-Valevski A. Myoclonic seizures as a main manifestation of Epstein-Barr virus infection. J Child Neurol. (2002) 17:446–7. 10.1177/088307380201700609 12174966

[32] Foss HD Herbst H Hummel M Araujo I Latza U Rancsò C. Patterns of cytokine gene expression in infectious mononucleosis. Blood (1994) 83:707–12. 8298133

[33] Hadinoto V Shapiro M Greenough TC Sullivan JL Luzuriaga K Thorley-Lawson DA. On the dynamics of acute EBV infection and the pathogenesis of infectious mononucleosis. Blood (2008) 111:1420–7. 10.1182/blood-2007-06-093278 17991806 PMC2214734

[34] Hadinoto V Shapiro M Greenough TC Sullivan JL Luzuriaga K Thorley-Lawson DA Digital droplet PCR (ddPCR) for the precise quantification of human T-lymphotropic virus 1 proviral loads in peripheral blood and cerebrospinal fluid of HAM/TSP patients and identification of viral mutations. J Neurovirol. (2014) 20:341–51. 10.1007/s13365-014-0249-3 24781526 PMC4085507

[35] Yoshikawa T Kato Y Ihira M Nishimura N Ozaki T Kumagai T. Kinetics of cytokine and chemokine responses in patients with primary human herpesvirus 6 infection. J Clin Virol. (2011) 50:65–8. 10.1016/j.jcv.2010.09.017 21035385

[36] Ward KN Gray JJ. Primary human herpesvirus-6 infection is frequently overlooked as a cause of febrile fits in young children. J Med Virol. (1994) 42:119–23. 10.1002/jmv.1890420204 8158105

[37] Barone SR Kaplan MH Krilov LR. Human herpesvirus-6 infection in children with first febrile seizures. J Pediatr. (1995) 127:95–7. 10.1016/S0022-3476(95)70263-6 7608818

[38] Asano Y Yoshikawa T Suga S Kobayashi I Nakashima T Yazaki T. Clinical features of infants with primary human herpesvirus 6 infection (exanthem subitum, roseola infantum). Pediatrics (1994) 93:104–8. 8265302

[39] Bertolani MF Portolani M Marotti F Sabbattini AM Chiossi C Bandieri MR. A study of childhood febrile convulsions with particular reference to HHV-6 infection: Pathogenic considerations. Childs Nerv Syst. (1996) 12:534–9. 10.1007/BF00261607 8906369

[40] Hall CB Long CE Schnabel KC Caserta MT McIntyre KM Costanzo MA. Human herpesvirus-6 infection in children. A prospective study of complications and reactivation. N Engl J Med. (1994) 331:432–8. 10.1056/NEJM199408183310703 8035839

[41] Clark DA Kidd IM Collingham KE Tarlow M Ayeni T Riordan A. Diagnosis of primary human herpesvirus 6 and 7 infections in febrile infants by polymerase chain reaction. Arch Dis Child. (1997) 77:42–5. 10.1136/adc.77.1.42 9279150 PMC1717251

[42] Jarrett RF Clark DA Josephs SF Onions DE. Detection of human herpesvirus-6 DNA in peripheral blood and saliva. J Med Virol. (1990) 32:73–6. 10.1002/jmv.1890320113 2173740

[43] Cone RW Huang ML Ashley R Corey L. Human herpesvirus 6 DNA in peripheral blood cells and saliva from immunocompetent individuals. J Clin Microbiol. (1993) 31:1262–7. 8388889 10.1128/jcm.31.5.1262-1267.1993 PMC262915

[44] Descamps V Avenel-Audran M Valeyrie-Allanore L Bensaid B Barbaud A Al Jawhari M. Saliva polymerase chain reaction assay for detection and follow-up of herpesvirus reactivation in patients with drug reaction with eosinophilia and systemic symptoms (DRESS). JAMA Dermatol. (2013) 149:565–9. 10.1001/jamadermatol.2013.2018 23426332

[45] Kasolo FC Mpabalwani E Gompels UA. Infection with AIDS-related herpesviruses in human immunodeficiency virus-negative infants and endemic childhood Kaposi's sarcoma in Africa. J Gen Virol. (1997) 78:847–55. 9129658 10.1099/0022-1317-78-4-847

[46] Soldan SS Berti R Salem N Secchiero P Flamand L Calabresi PA. Association of human herpes virus 6 (HHV-6) with multiple sclerosis: increased IgM response to HHV-6 early antigen and detection of serum HHV-6 DNA. Nat Med. (1997) 3:1394–7. 9396611 10.1038/nm1297-1394

[47] Crawford JR Kadom N Santi MR Mariani B Lavenstein BL. Human herpesvirus 6 rhombencephalitis in immunocompetent children. J Child Neurol. (2007) 22:1260–8. 10.1177/0883073807307086 18006954

[48] Bale JF. Human herpesviruses and neurological disorders of childhood. Semin Pediatr Neurol. (1999) 6:278–87. 10.1016/S1071-9091(99)80026-6 10649836

[49] Getts DR Balcar VJ Matsumoto I Müller M King NJ. Viruses and the immune system: their roles in seizure cascade development. J Neurochem. (2008) 104:1167–76. 10.1111/j.1471-4159.2007.05171.x 18205751

[50] Ejme J Infectious mononucleosis. Acta Med Scand. (1964) 413 (Suppl):1–83. 14154704

[51] Bathoorn E Vlaminckx BJ Schoondermark-Stolk S Donders R van der Meulen M Thijsen SF. Primary Epstein-Barr virus infection with neurological complications. Scand J Infect Dis. (2011) 43:136–44. 10.3109/00365548.2010.531760 21070089

[52] Levin LI Munger KL Rubertone MV Peck CA Lennette ET Spiegelman D. Temporal relationship between elevation of epstein-barr virus antibody titers and initial onset of neurological symptoms in multiple sclerosis. JAMA (2005) 293:2496–500. 10.1001/jama.293.20.2496 15914750

[53] Svahn A Berggren J Parke A Storsaeter J Thorstensson R Linde A. Changes in seroprevalence to four herpesviruses over 30 years in Swedish children aged 9–12 years. J Clin Virol. (2006) 37:118–23. 10.1016/j.jcv.2006.07.012 16971177

[54] Niederman JC Evans AS Subrahmanyan L McCollum RW. Prevalence, incidence and persistence of EB virus antibody in young adults. N Engl J Med. (1970) 282:361–5. 10.1056/NEJM197002122820704 4312365

[55] Niederman JC Evans AS Subrahmanyan L McCollum RW Clinical and virologic manifestations of primary Epstein-Barr virus (EBV) infection in Kenyan infants born to HIV-infected women. J Infect Dis. (2013) 207:1798–806. 10.1093/infdis/jit093 23493724 PMC3654744

[56] Xiong ZQ Qian W Suzuki K McNamara JO. Formation of complement membrane attack complex in mammalian cerebral cortex evokes seizures and neurodegeneration. J. Neurosci. (2003) 23:955–60. 10.1523/JNEUROSCI.23-03-00955.2003 12574424 PMC6741927

[57] Long Q Upadhya D Hattiangady B Kim DK An SY Shuai B. Intranasal MSC-derived A1-exosomes ease inflammation, and prevent abnormal neurogenesis and memory dysfunction after status epilepticus. Proc Natl Acad Sci USA. (2017) 114:E3536–45. 10.1073/pnas.1703920114 28396435 PMC5410779

[58] Dupuis N Mazarati A Desnous B Chhor V Fleiss B Le Charpentier T. Pro-epileptogenic effects of viral-like inflammation in both mature and immature brains. J Neuroinflamm. (2016) 13:307. 10.1186/s12974-016-0773-6 27955671 PMC5153898

[59] Virta M Hurme M Helminen M. Increased plasma levels of pro- and anti-inflammatory cytokines in patients with febrile seizures. Epilepsia (2002) 43:920–3. 10.1046/j.1528-1157.2002.02002.x 12181012

[60] Ichiyama T Nishikawa M Yoshitomi T Hayashi T Furukawa S Tumor necrosis factor-α, interleukin-1β, and interleukin-6 in cerebrospinal fluid from children with prolonged febrile seizures. Comparison with acute encephalitis/encephalopathy. Neurology (1998) 50:407–11. 10.1212/WNL.50.2.407 9484363

[61] Dubé C Vezzani A Behrens M Bartfai T Baram TZ. Interleukin-1β contributes to the generation of experimental febrile seizures. Ann Neurol. (2005) 57:152–5. 10.1002/ana.20358 15622539 PMC2909879

[62] Marchi N Fan Q Ghosh C Fazio V Bertolini F Betto G. Antagonism of peripheral inflammation reduces the severity of status epilepticus. Neurobiol Dis. (2009) 33:171–81. 10.1016/j.nbd.2008.10.002 19010416 PMC3045783

[63] Fukuda M Hino H Suzuki Y Takahashi H Morimoto T Ishii E. Postnatal interleukin-1β enhances adulthood seizure susceptibility and neuronal cell death after prolonged experimental febrile seizures in infantile rats. Acta Neurol Belg. (2014) 114:179–85. 10.1007/s13760-013-0246-y 24002650

[64] Riis JL Out D Dorn LD Beal SJ Denson LA Pabst S. Salivary cytokines in healthy adolescent girls: intercorrelations, stability, and associations with serum cytokines, age, and pubertal stage. Dev Psychobiol. (2014) 56:797–811. 10.1002/dev.21149 23868603 PMC8136359

[65] Berdat PA Wehrle TJ Küng A Achermann F Sutter M Carrel TP. Age-specific analysis of normal cytokine levels in healthy infants. Clin Chem Lab Med. (2003) 41:1335–9. 10.1515/CCLM.2003.204 14580162

[66] Kim HO Kim HS Youn JC Shin EC Park S. Serum cytokine profiles in healthy young and elderly population assessed using multiplexed bead-based immunoassays. J Transl Med. (2011) 9:113. 10.1186/1479-5876-9-113 21774806 PMC3146842

[67] Stowe RP Peek MK Cutchin MP Goodwin JS. Plasma cytokine levels in a population-based study: relation to age and ethnicity. J Gerontol A Biol Sci Med Sci. (2010) 65:429–33. 10.1093/gerona/glp198 20018825 PMC2844059

[68] Böhler T Canivet C Nguyen PN Galvani S Thomsen M Durand D. Cytokines correlate with age in healthy volunteers, dialysis patients and kidney-transplant patients. Cytokine (2009) 45:169–73. 10.1016/j.cyto.2008.11.014 19147373

