# Supplementary material for: Detection of HHV-6 and EBV and Cytokine Levels in Saliva From Children With Seizures: Results of a Multi-Center Cross-Sectional Study
Source: Front Neurol. 2018 Oct 5;9:834. doi: 10.3389/fneur.2018.00834 (PMC6182262; doi:10.3389/fneur.2018.00834)
Supplement: Supplementary file 1 [file Data_Sheet_1.docx]

**Appendix**

**Supplementary Table 1. Internal validation of viral DNA detection by means of droplet digital PCR comparing the use of swab vs. mouth rinse in 10 healthy adult volunteers.**

|  | Swab (copies/mL) | | Mouth rinse (copies/mL) | |
| --- | --- | --- | --- | --- |
| HV identifier | EBV | HHV-6B | EBV | HHV-6B |
| 1 | 0 | 0 | 0 | 0 |
| 2 | 53625 | 487 | 160875 | 650 |
| 3 | 0 | 650 | 0 | 1787 |
| 4 | 0 | 0 | 0 | 0 |
| 5 | 1787 | 0 | 1300 | 0 |
| 6 | 2600 | 0 | 5037 | 1137 |
| 7 | 0 | 487 | 0 | 0 |
| 8 | 0 | 650 | 0 | 0 |
| 9 | 0 | 0 | 3412 | 0 |
| 10 | 0 | 0 | 1137 | 0 |

HV, healthy volunteer
